# UCA1 promotes cell proliferation and invasion of gastric cancer by targeting CREB1 sponging to miR‐590‐3p

**DOI:** 10.1002/cam4.1310

**Published:** 2018-03-08

**Authors:** Lei Gu, Lie‐sheng Lu, Dong‐lei Zhou, Zhong‐chen Liu

**Affiliations:** ^1^ Department of General Surgery Shanghai Tenth People's Hospital School of Medicine Tongji University Shanghai China

**Keywords:** CREB1, gastric cancer, miR‐590‐3p, UCA1

## Abstract

Long noncoding RNAs (lncRNAs) have emerged as regulators in a variety of biological processes, including carcinogenesis in human cancer. UCA1 has been reported to be upregulated in gastric cancer (GC); however, the underlying functional roles of UCA1 in GC have not been established. In the current study, we showed that UCA1 is significantly higher in GC tissues and cells compared with adjacent normal tissues and a gastric epithelium cell line, respectively. Higher UCA1 expression was associated with lymph node metastasis, TNM stage, and poor overall survival (OS) in GC patients. In vitro functional studies confirmed that UCA1 promotes cell proliferation, colony formation ability, and cell invasion in GC cells. We demonstrated that knockdown of UCA1 inhibits tumor growth in vivo. The double luciferase reporter, RNA‐binding protein immunoprecipitation assay, and RNA pull down assay demonstrated that miR‐590‐3p serves as a target for UCA1. UCA1 promoted cell proliferation and invasion by negatively regulating miR‐590‐3p expression. Moreover, we demonstrated that CREB1 is a downstream target of miR‐590‐3p and UCA1 activates CREB1 expression by sponging to miR‐590‐3p. Thus, these results showed that UCA1 functions as an oncogene in GC and may be a target for treatment of GC.

## Introduction

Gastric cancer (GC) represents a large threat to public health with a high incidence and mortality rate worldwide. Recently, despite the large advances in diagnostic and therapeutic approaches, including surgical methods, radiotherapy, chemotherapy, and novel molecular targeted therapy for GC, the 5‐year survival rate for patients who had been diagnosed in an advanced stage is poor 1, 2. Thus, the molecular mechanisms underlying GC progression is in need of continued investigation to provide promising therapeutic targets.

Accumulating evidence has highlighted that long noncoding RNAs (lncRNAs) play crucial roles in a variety of biological processes, including cell differentiation, proliferation, and apoptosis. Dysregulated expression of lncRNAs has been confirmed to be involved in GC development and progression 3, 4. The lncRNA, urothelial carcinoma‐associated 1 (UCA1), has been identified as an oncogene that enhances cell proliferation, inhibits apoptosis, and promotes cell cycle progression in some tumors 5. Yang et al. 6 reported that UCA1 promotes the progression of oral squamous cell carcinoma by activating the WNT/*β*‐catenin signaling pathway. Xiao et al. 7 demonstrated that UCA1 promotes epithelial‐mesenchymal transition (EMT) of breast cancer cells by enhancing the Wnt/beta‐catenin signaling pathway. UCA1 promotes the progression and regulates proliferation through the KLF4‐KRT6/13 signaling pathway in prostate cancer 8. UCA1 has been shown to be a novel diagnostic and predictive biomarker in plasma for early GC 9. TGF*β*1 induces the upregulation of UCA1, which promotes invasion and migration in GC 10.

In the current study, we demonstrated that UCA1 is increased in GC tissues and cells. UCA1 promoted GC cell growth in vitro and in vivo. Furthermore, we demonstrated that UCA1 inhibit CREB1 expression by sponging to miR‐590‐3p in GC cells. Thus, UCA1 functions as an oncogene and may be a target for GC treatment.

## Materials and Methods

### Patient tissue samples

We obtained 62 GC tissue samples and matched adjacent normal tissues from patients who underwent surgical resection in the Department of General Surgery of Shanghai Tenth People's Hospital (School of Medicine, Tongji University). After surgical resection, tissues samples were immediately snap‐frozen in liquid nitrogen, then stored at −80°C for further analysis. The study conformed to the standards set by the Declaration of Helsinki. No radiotherapy or chemotherapy was administered before surgery. Written informed consent was collected from all patients. This study was approved by the Institutional Ethical Board of Shanghai Tenth People's Hospital.

### Cell cultures

Four human GC cell lines (AGS, MKN‐28, SGC‐7901, and MKN‐45) and a normal gastric epithelium cell line (GES‐1) were purchased from the Institute of Biochemistry and Cell Biology of the Chinese Academy of Sciences (Shanghai, China). Cells were cultured in RPMI ‐1640 (FBS, Gibco, Thermo Scientific, Waltham) and supplemented with 10% fetal bovine serum (FBS, Gibco, Thermo Scientific). Cells were cultured in a humidified incubator at 37°C in the presence of 5% CO_2_.

### Cell transfection

The siRNAs were transfected into cells, using Lipofectamine 2000. The two siRNAs against UCA1 were purchased from Ribobio (Guangzhou, China). The pcDNA3.1‐UCA1 was constructed by chemical synthesis of full‐length sequences, then cloned into the Hind III/EcoR I sites of pcDNA3.1 by Ribobio.

### Quantitative real‐time reverse transcription PCR

Total RNA was extracted using TRIzol reagent (Invitrogen, CA) from GC tissues and cells according to the manufacturer's protocol. The RNA was reverse‐transcribed into cDNA using PrimeScript RT Reagent (TaKaRa, Dalian, China).The levels of mRNA expression were detected using a SYBR‐Green PCR Master Mix Kit (TaKaRa) and performed on a 7500 System (Applied Biosystems, Carlsbad, CA). The primer sequences were as follows: UCA1 forward, 5'‐TTTGCCAGCCTCAGCTTAAT‐3', UCA1 reverse, 5'‐TTGTCCCCATTTTCCATCAT‐3'; GAPDH forward, 5'‐CCACCCATGGCAAATTCCATGGCA‐3'; and GAPDH reverse, 5'‐TCTAGACGGCAGGTCAGGTCCACC‐3'.

### Cell proliferation assay

The MTT assay was applied to assess cell proliferation ability. Transfected cells (3000 cells/well) were seeded into 96‐well plates, and 20 *μ*L of the MTT solution (5 mg/mL) was added to each well for 4 h. Cell proliferation ability was measured daily for 5 days. The absorbance was read at 490 nm on a micro‐plate reader (Bio Tek Instruments, Inc., Winooski, VT). For the cell colony formation assay, the transfected cells (300 cells/well) were seeded into 6‐well plates, and cells were cultured in a humidified incubator at 37°C in the presence of 5% CO_2_. After 14 days, cells were fixed with methanol and stained with 1% crystal violet for 15 min. Then, the number of cell colonies was counted.

### Cell invasion assay

The cell invasion assay was performed as previously described 6. A total of 3 × 10^5^ transfected cells in 100 *μ*L of serum‐free RPMI‐1640 medium were added onto Transwell upper chambers (Corning, city, NY). Five hundred microliters of RPMI‐1640 medium with 10% FBS were added onto Transwell lower chambers. After cells were cultured for 48 h, the membranes were fixed with methanol and stained using 0.1% crystal violet. Finally, the invasive cells were counted in 10 random fields.

### Western blot analysis

Cells were lysed using RIPA buffer (Beyotime, Shanghai, China). The protein concentration was determined using a BCA protein assay kit (Beyotime). An equal amount of protein was added to SDS‐PAGE and transferred to polyvinylidene fluoride (PVDF) membranes. Then, the membranes were blocked with 5% nonfat dry milk. The primary antibodies used in the study included CREB1 (1: 2000; Abcam, USA) and GAPDH (1: 1000; Abcam, Cambridge, MA, USA). Furthermore, the membranes were incubated with HRP‐conjugated secondary antibodies for 2 h. Protein blots were visualized, using an enhanced chemiluminescence (ECL) detection system.

### Dual luciferase reporter assay

The sequences corresponding to UCA1 mRNA and containing the wild‐type or mutated miR‐590‐3p binding sequence or the 3'‐UTR of CREB1 mRNA and containing the wild‐type or mutated miR‐590‐3p binding sequence were synthesized by Ribobio. The above sequences were subcloned into the pmiR‐GLo luciferase reporter vector (Promega, Madison, WI). Cells were co‐transfected with pmiR‐GLO‐UCA1‐WT or pmiR‐GLO‐UCA1‐MUT reporter plasmids together with miR‐590‐3p mimic or negative control, or co‐transfected with the pmiR‐GLO‐3'‐UTR‐CREB1‐WT or pmiR‐GLO‐3'‐UTR‐CREB1‐MUT reporter plasmids together with miR‐590‐3p mimic, si‐UCA1, or negative control oligoribonucleotides using Lipofectamine 2000. Firefly and Renilla luciferase activities were detected, using a dual luciferase reporter system.

### RNA‐binding protein immunoprecipitation assay

A RNA‐binding protein immunoprecipitation (RIP) assay was performed using the EZ‐Magna RIP Kit (Millipore, Billerica, MA) according to the manufacturer's instructions. Magnetic beads conjugated with human Ago2 antibody (Millipore) or negative control mouse IgG (CST, USA) was added to the RIP buffer. The mRNA of UCA1 was detected by Quantitative real‐time reverse transcription PCR **(**qRT‐PCR).

### RNA pull‐down assay

The RNA pull‐down assay was performed as previously described 11. Cells lysates were incubated with streptavidin‐coated magnetic beads containing bio‐miR‐590‐3p‐wt, bio‐miR‐590‐3p‐mut, or bio‐miRNA negative control (Bio‐miR‐NC; Life Technologies, Waltham, MA). UCA1 expression was determined by qRT‐PCR.

### Tumor formation in nude mice

SGC‐7901 cells were transfected with Lenti‐control or Lenti‐shRNA‐UCA1. Cells (2 × 10^6^ cells) were subcutaneously injected into 3‐week‐old BALB/c nude mice. After 4 weeks, the tumors were excised. Tumor volume was detected every week after injection. The tumor tissues were used for further analysis. This study was approved by the Institutional Committee for Animal Research.

### Statistical analysis

The data were analyzed using an independent *t*‐test (SPSS, Inc., Chicago, IL) with Student's *t*‐test. A *P* < 0.05 was considered statistically significant. All of the experiments were repeated 3 times. The data are presented as the mean ± SD.

## Results

### Expression of UCA1 is higher in GC tissues and cells

**Figure 1:**
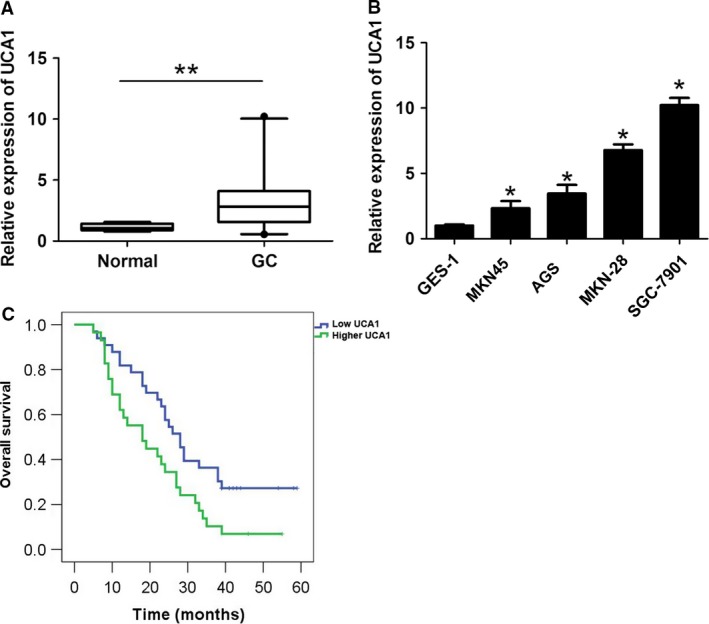
UCA1 was significantly upregulated in GC and associated with shorter survival time of GC patients. (A) The UCA1 expression was upregulated in GC tissues compared with adjacent normal tissues. **P < 0.01 as determined by Student's *t*‐test. (B) UCA1 was upregulated in GC cells (AGS, MKN‐45, MKN‐28, and SGC‐7901) compared with GES‐1 cells. (C) Higher UCA1 expression was associated with shorter survival time compared with lower UCA1 expression in GC patients. *P < 0.05. GC, gastric cancer.

To determine the expression of UCA1 in GC tissues and adjacent normal tissues, we performed qRT‐PCR. As shown in Figure 1A, the expression of UCA1 in GC tissues was higher when compared with adjacent normal tissues. We also analyzed the expression of UCA1 in four human GC cell lines (AGS, MKN‐45, SGC‐7901, and MKN‐28) and a normal gastric epithelium cell line (GES‐1). The statistical analysis demonstrated that UCA1 expression was higher in GC cells when compared with GES‐1 cells (Fig. 1B). Furthermore, the relationship between UCA1 and clinicopathologic factors verified that UCA1 expression was positively associated with lymph node metastasis and TNM stage in patients (*P* < 0.05, Table 1). Compared with the lower UCA1 expression group, Kaplan‐Meier curve analysis showed that the higher UCA1 expression group was associated with a worse survival time in GC patients (Fig. 1C).

**Table 1:** Correlation between UCA expression and clinicopathologic factors of 62 GC, gastric cancer patients

Factors	Patient number	UCA1 expression		*P*‐value
		Lower	Higher	
Gender				0.297
Male	43	21	22	
Female	19	12	7	
Age(years)				0.200
≤50	42	20	22	
>50	20	13	7	
Local invasion				0.665
T1,T2	36	20	16	
T3,T4	26	13	13	
Differentiation				0.458
High, middle	39	22	17	
Low	23	11	12	
Lymph node metastasis				0.003^a^
Negative	34	24	10	
Positive	28	9	19	
TNM stage				0.009^a^
I‐II	34	23	11	
III‐IV	28	10	18	

a *P* < 0.05

### UCA1 promotes cell proliferation and cell invasion of GC

**Figure 2:**
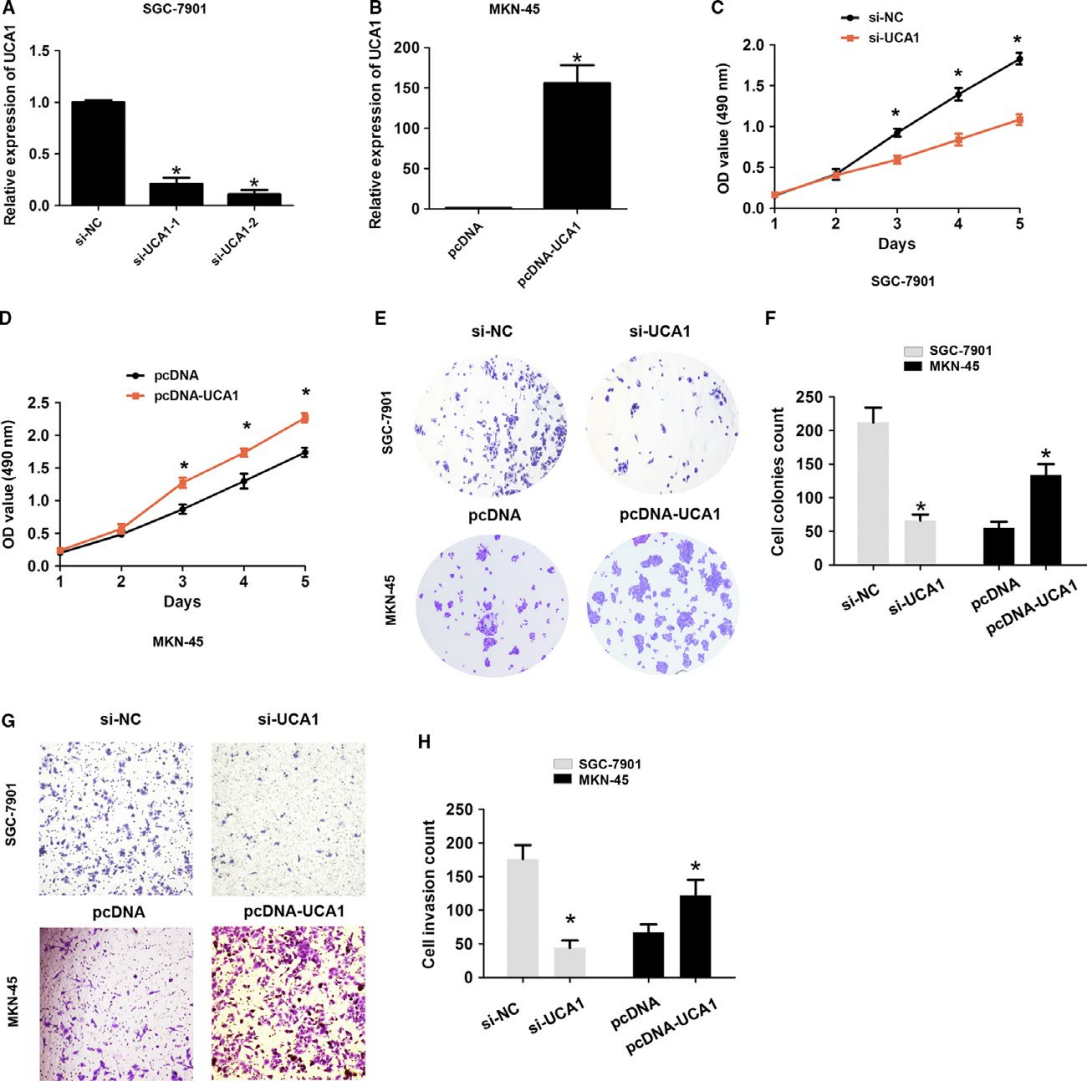
UCA1 promoted cell proliferation and cell invasion of GC. (A–B) UCA1 expression was detected by transfection with si‐NC, si‐UCA1‐1, and si‐UCA1‐2 into SGC‐7901 cells or transfection with pcDNA or pcDNA‐UCA1 into MKN‐45 cells. (C–D) A MTT assay was performed by transfection with si‐NC and si‐UCA1 into SGC‐7901 cells or transfection with pcDNA and pcDNA‐UCA1 into MKN‐45 cells after cell transfection at 1, 2, 3, 4, and 5 days. (E–F) A cell colony formation assay was performed by transfection with si‐NC and si‐UCA1 into SGC‐7901 cells or transfection with pcDNA and pcDNA‐UCA1 into MKN‐45 cells. (G–H) A cell invasion assay were performed and the cell number was analyzed by transfection with si‐NC and si‐UCA1 into SGC‐7901 cells or transfection with pcDNA and pcDNA‐UCA1 into MKN‐45 cells. The data are presented as the mean ± SD from three independent experiments. *P < 0.05; GC, gastric cancer.

To analyze the effects of UCA1 on the proliferation and invasion of GC, we used siRNA‐UCA1 or pcDNA3.1‐UCA1 plasmid for silencing and overexpression of UCA1 in SGC‐7901 and MKN‐45 cells (Fig. 2A–B). The siRNA‐2 was applied for UCA1 silencing in the following experiments based on a higher knockdown efficiency of UCA1 in SGC‐7901 cells. MTT assays observed that knockdown of UCA1 inhibited the cell proliferation rate in SGC‐7901 cells; however, upregulation of UCA1 enhanced the cell proliferation rate in MKN‐45 cells (Fig. 2C–D). The cell colony assay results showed that cell colonies were smaller after UCA1 silencing in SGC‐7901 cells, but larger after upregulation of UCA1 in MKN‐45 cells (Fig. 2E–F). Moreover, cell invasion ability was suppressed after knockdown of UCA1 in SGC‐7901 cells, which was enhanced after upregulation of UCA1 in MKN‐45 cells (Fig. 2G–H). Thus, these data demonstrated that UCA1 promotes cell proliferation and cell invasion of GC.

### MiR‐590‐3p is a target of UCA1 in GC cells

**Figure 3:**
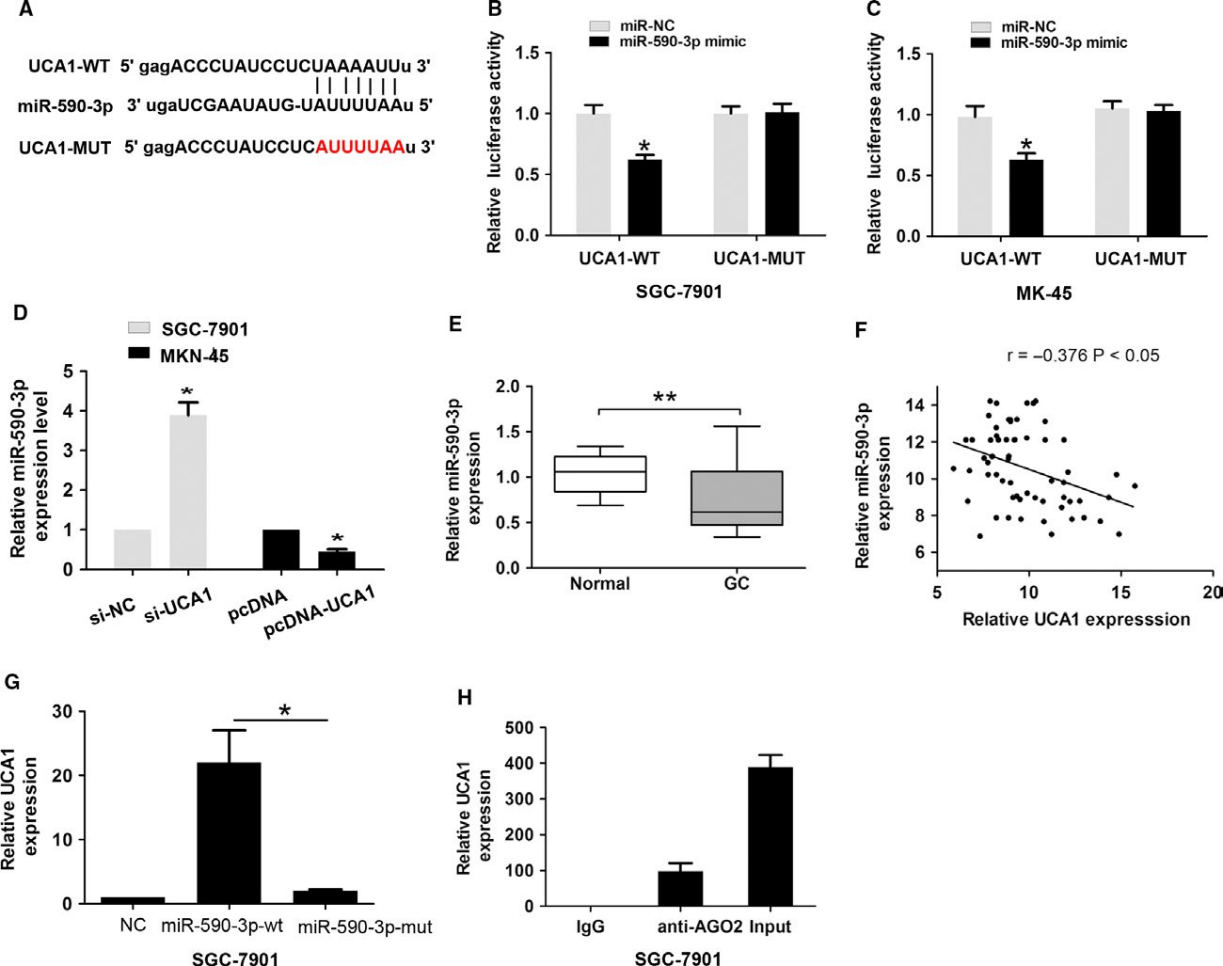
UCA1 acted as a molecular sponge for miR‐590‐3p in GC cells. (A) Alignment of potential UCA1 base pairing with miR‐590‐3p was predicted by miRanda. (B–C) The relative luciferase activities were detected by transfecting pmirGlo‐UCA1‐WT or pmirGLo‐UCA1‐MUT and miR‐590‐3p or miR‐NC into SGC‐7901 and MKN‐45 cells. (D) The relative expression of miR‐590‐3p was measured after knockdown of UCA1 in SGC‐7901 cells or overexpression of UCA1 in MKN‐45 cells by qRT‐PCR. (E) The relative expression of miR‐590‐3p was measured in GC tissues when compared with adjacent normal tissues by qRT‐PCR. (F) The correlation between UCA1 and miR‐590‐3p level was measured in 62 GC tissues by Pearson correlation analysis. (G) RNA pull‐down assay with biotinylated‐NC, biotinylated miR‐590‐3p‐wt, and biotinylated miR‐590‐3p‐mut probe. The relative expression of UCA1 was measured by qRT‐PCR. (H) RNA‐binding protein immunoprecipitation experiment using negative control (mouse IgG) and Ago2 antibody was performed. The relative expression of UCA1 was measured by qRT‐PCR. The data are presented as the mean ± SD from three independent experiments.*P < 0.05, **P < 0.01; GC, gastric cancer by qRT‐PCR, Quantitative real‐time reverse transcription PCR.

Competing endogenous RNAs (ceRNAs) facilitate crosstalk among lncRNAs, mRNAs, and their shared miRNAs 12. Long noncoding RNAs can act as sinks for pools of active miRNAs to regulate target mRNAs 13. To determine whether or not UCA1 acts as a ceRNA in GC, we used a target prediction software (miRanda; http://www.micro-RNA.org/) to predict potential UCA1–miRNA interactions. As shown in Figure 3A, the predicted results showed that miR‐590‐3p had a potential binding site with UCA1. To further explore the mechanism by which UCA1 regulated miR‐590‐3p expression in GC, we arranged luciferase assays to detect the association between UCA1 and miR‐590‐3p. Luciferase reporter gene vectors containing UCA1‐WT and UCA1‐MUT binding sites with miR‐590‐3p were constructed (Fig. 3A). The SGC‐7901 and MKN‐45 cells were co‐transfected with UCA1‐WT and UCA1‐MUT luciferase reporter gene vectors and miR‐590‐3p mimic or miR‐NC. By performing dual luciferase assays, we found that miR‐590‐3p mimic significantly reduced the luciferase activity of the UCA1‐WT reporter gene vector, but there was no significant change in luciferase activity of the UCA1‐MUT reporter gene vector in SGC‐7901 and MKN‐45 cells (Fig. 3B–C). Furthermore, we found that miR‐590‐3p was significantly increased after knockdown of UCA1 in SGC‐7901 cells, but was down‐regulated after overexpression of UCA1 in MKN‐45 cells (Fig. 3D). Compared with adjacent normal tissues, miR‐590‐3p expression was significantly lower in GC tissues (Fig. 3E). Higher UCA1 expression had a negative association with lower miR‐590‐3p in GC tissues (*r* = −0.376, *P* < 0.05, Fig. 3F).

In addition, we performed a RNA‐pull down assay using biotinylated‐NC, biotinylated miR‐590‐3p‐wt, and biotinylated miR‐590‐3p‐mut probe in SGC‐7901 cells. UCA1 was pulled down by biotinylated miR‐590‐3p‐wt; however, UCA1 was not pulled down by the biotinylated miR‐590‐3p‐mut binding site between UCA1 and miR‐590‐3p (Fig. 3G). Ago2 protein is a key constituent of the RNA‐induced silencing complex (RISC), which acts as the mechanism underlying miRNA silencing of target genes 14. To detect whether or not UCA1 and miR‐590‐3p were present in the RISC complex, we performed a RIP experiment using Ago2 antibody. UCA1 was in the Ago2 pellet (Fig. 3H). Thus, miR‐590‐3p was a direct target of UCA1 in GC.

### UCA1 promotes cell proliferation and invasion of GC by regulating miR‐590‐3p in vitro

**Figure 4:**
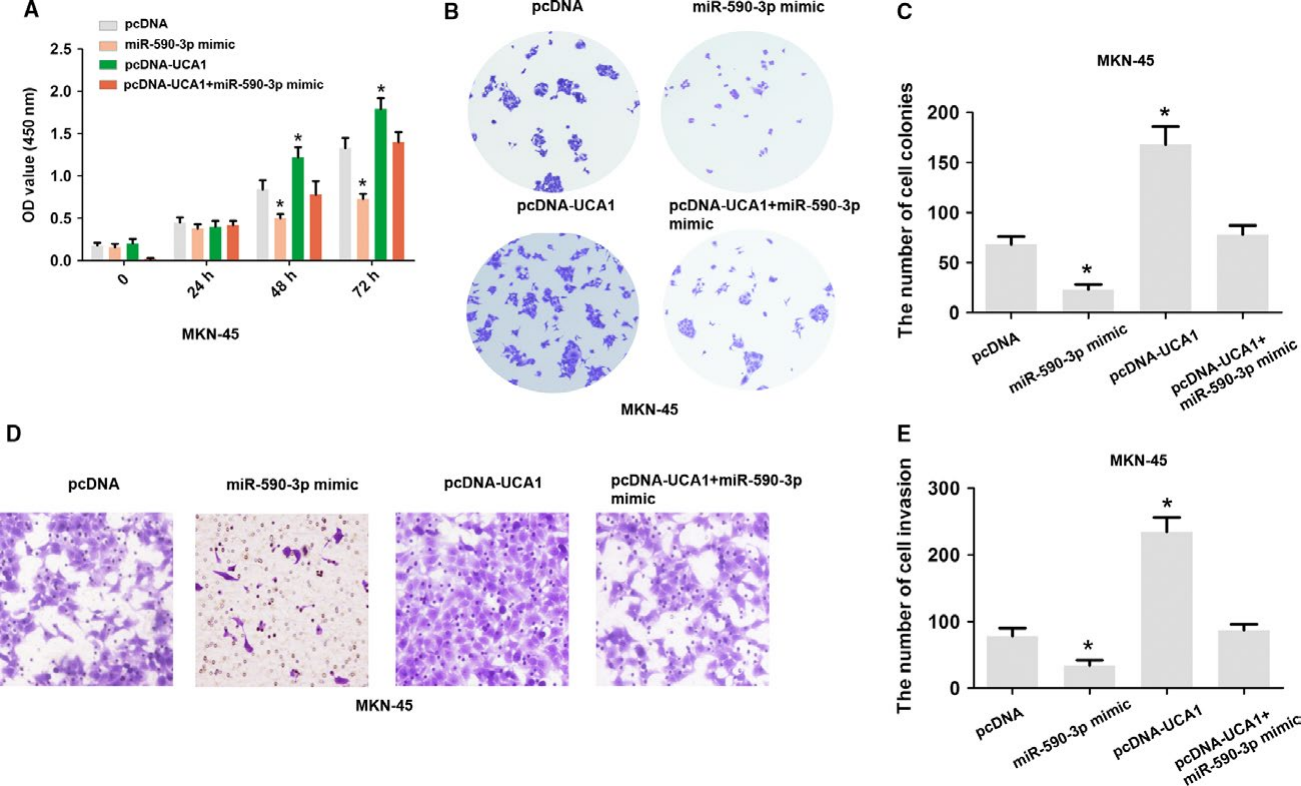
UCA1 promoted cell proliferation and invasion by regulating miR‐590‐3p. (A) A MTT assay was performed by transfection with pcDNA, pcDNA‐UCA1, miR‐590‐3p mimic, or pcDNA‐UCA1 and miR‐590‐3p mimic into MKN45 cells after cell transfection at 0, 24, 48, and 72 h. (B–C) A cell colony formation assay was performed and the number of colonies was analyzed by transfection with pcDNA, pcDNA‐UCA1, miR‐590‐3p mimic, or pcDNA‐UCA1 and miR‐590‐3p mimic into MKN45 cells. (D–E) A cell invasion assay was performed and the number of cells was analyzed by transfection with pcDNA, pcDNA‐UCA1, miR‐590‐3p mimic, or pcDNA‐UCA1 and miR‐590‐3p mimic in MKN45 cells. The data are presented as the mean ± SD from three independent experiments. *P < 0.05.

Furthermore, MTT cell proliferation and cell colony assays demonstrated that upregulation of UCA1 promotes cell proliferation and the number of cell colonies by transfecting pcDNA‐UCA1 plasmid into MKN45 cells, while the effects of co‐transfecting mir‐590‐3p mimic and pcDNA‐UCA1 induced by UCA1 overexpression were reversed (Fig. 4A–C). Moreover, cell invasion ability was enhanced by transfecting pcDNA‐UCA1 plasmid into MKN45 cells, while the effects of co‐transfecting mir‐590‐3p mimic and pcDNA‐UCA1 induced by UCA1 silencing were dismissed (Fig. 4D–E). Together, these results showed that UCA1 promotes cell proliferation and invasion by regulating miR‐590‐3p in GC.

### UCA1 promotes CREB1 expression by regulating miR‐590‐3p in GC

**Figure 5:**
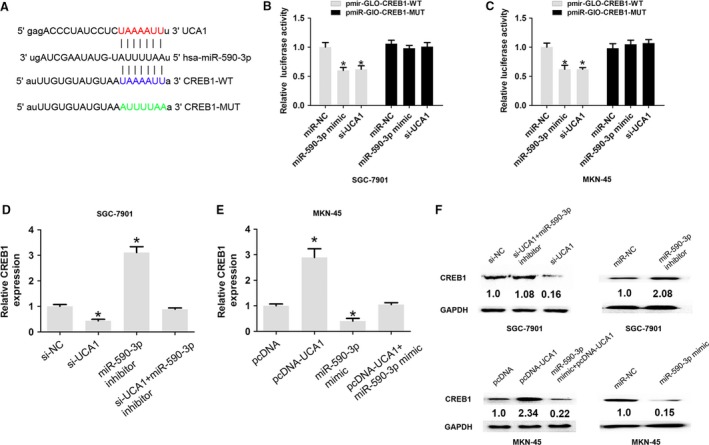
UCA1 promoted CREB1 expression by regulating miR‐590‐3p expression in GC cells. (A) Alignment of potential CREB1 base pairing with miR‐590‐3p was predicted by miRanda. (B–C) The relative luciferase activities were detected by transfected with miR‐NC, miR‐590‐3p mimic, si‐UCA1, and pmirGlo‐3'UTR‐CREB1‐WT or pmirGlo‐3'UTR‐CREB1‐MUT into SGC‐7901 and MKN‐45 cells. (D) The relative mRNA expression of CREB1 was detected by transfecting with si‐NC, si‐UCA1, miR‐590‐3p inhibitor, or si‐UCA1 and miR‐590‐3p inhibitor in SGC‐7901 cells. (E) The relative mRNA expression of CREB1 was analyzed by transfection with pcDNA, pcDNA‐UCA1, miR‐590‐3p mimic, or pcDNA‐UCA1 + miR‐590‐3p mimic in MKN‐45 cells. (F) The relative protein expression of CREB1 was analyzed by transfecting with transfecting with si‐NC, si‐UCA1, miR‐590‐3p inhibitor, or si‐UCA1 and miR‐590‐3p inhibitor into SGC‐7901 cells or transfecting with pcDNA, pcDNA‐UCA1, miR‐590‐3p mimic, or pcDNA‐UCA1 + miR‐590‐3p mimic into MKN‐45 cells. The data are presented as mean ± SD from three independent experiments.*P < 0.05.

MiR‐590‐3p had been shown to be a tumor suppressor in some tumors 15. Using prediction tools (miRanda and Target Scan), we showed that CREB1 has a potential binding site with miR‐590‐3p. Luciferase reporter gene vectors containing 3'UTR‐CERB1‐WT and 3'UTR‐CERB1‐MUT binding sites with miR‐590‐3p were constructed (Fig. 5A). The SGC‐7901 and MKN‐45 cells were co‐transfected with 3'UTR‐CERB1‐WT and 3'UTR‐CERB1‐MUT luciferase reporter gene vectors and miR‐NC, miR‐590‐3p mimic, or si‐UCA1. By performing dual luciferase assays, we found that miR‐590‐3p mimic significantly reduced the luciferase activity of the 3'UTR‐CERB1‐WT reporter gene vector, but there was no significant change in luciferase activity of the 3'UTR‐CERB1‐MUT reporter gene vector in MKN‐45 cells. Moreover, si‐UCA1 significantly reduced the luciferase activity of the 3'UTR‐CERB1‐WT reporter gene vector, but there was no significant change in luciferase activity of the 3'UTR‐CERB1‐MUT reporter gene vector in SGC‐7901 and MKN‐45 cells (Fig. 5B–C).

We transfected si‐NC, si‐UCA1, miR‐590‐3p inhibitor, and si‐UCA1 + miR‐590‐3p inhibitor into SGC‐7901 cells, and the results showed that miR‐590‐3p inhibitor promoted the mRNA expression of CREB1 in SGC‐7901 cells, but co‐transfection with si‐UCA1 and miR‐590‐3p inhibitor dismissed the effects (Fig. 5D and F top). We transfected pcDNA, pcDNA‐UCA1, miR‐590‐3p mimic, and pcDNA‐UCA1 + miR‐590‐3p mimic into MKN45 cells, and the results showed that miR‐590‐3p mimic inhibited the mRNA expression of CREB1 in MKN‐45 cells, but co‐transfection with pcDNA‐UCA1 and miR‐590‐3p mimic dismissed the effects (Fig. 5E and F low). These results showed that UCA1 promotes CREB1 expression by regulating miR‐590‐3p in GC.

### Knockdown of UCA1 inhibits tumor growth by regulating miR‐590‐3p expression *in vivo*

**Figure 6:**
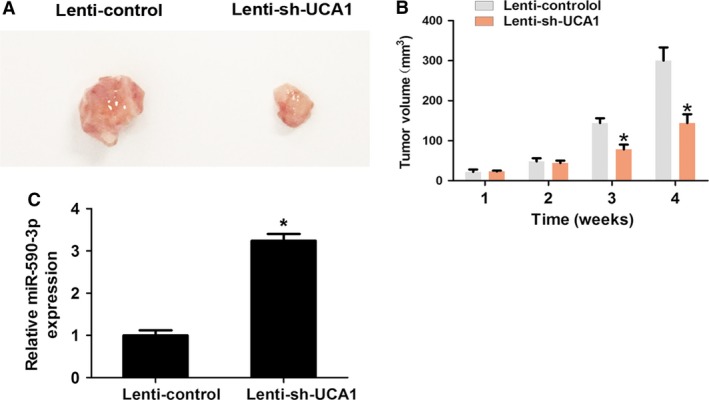
Knockdown of UCA1 inhibited tumor growth by regulating miR‐590‐3p expression in vivo. (A) Photographs of excised tumors 4 weeks postinjection were obtained. (B) The mean tumor volume was detected every week in nude mice injected with transfected Lenti‐control or Lenti‐shRNA‐UCA1. (C) MiR‐590‐3p was detected by quantitative real‐time reverse transcription PCR, in nude mice injected with transfected Lenti‐control or Lenti‐shRNA‐UCA1. *P < 0.05.

We further characterized whether or not UCA1 affected tumor growth *in vivo*. SGC‐7901 cells were transfected with Lenti‐control or Lenti‐shRNA‐UCA1. Cells (2 × 10^6^ cells) were subcutaneously injected into 3‐week‐old BALB/c nude mice. After 4 weeks, the tumors were excised. The results showed that knockdown of UCA1 inhibited tumor growth when compared with the control group (Fig. 6A–B). We also demonstrated that knockdown of UCA1 upregulated miR‐590‐3p in vivo (Fig. 6C). Thus, knockdown of UCA1 inhibited tumor growth by regulating miR‐590‐3p expression in vivo.

## Discussion

A number of studies have reported that lncRNAs correlate with the progression of GC. In previous reports, UCA1 had been identified as an oncogene in some cancer types. Upregulation of UCA1 promotes cell proliferation and invasion in hypopharyngeal carcinoma and predicts poor survival of hypopharyngeal carcinoma patients 16. Long noncoding RNA UCA1 expression in glioma samples is higher, promotes proliferation, and predicts poor prognosis in patients with gliomas 17. Knockdown of UCA1 inhibits cell proliferative activities, induces cell apoptosis, and causes cell cycle arrest in patients with pancreatic cancer 18. Upregulated lncRNA‐UCA1 contributes to progression of lung cancer and is closely related to clinical diagnosis as a predictive biomarker in plasma 19. In the current study, we showed that UCA1 was higher in GC tissues and cells compared with adjacent normal tissues and GES‐1 cell, respectively. Furthermore, we demonstrated that higher UCA1 expression is associated with lymph node metastasis, TNM stage, and worse OS in GC patients. We further investigated the functional effects of UCA1 on cell proliferation and invasion ability, and the results confirmed that UCA1 promotes cell proliferation, colony formation, and cell invasion in GC cells. We showed that knockdown of UCA1 inhibited tumor growth in vivo. Thus, our results indicated that UCA1 acts as an oncogene, which is consistent with previous studies; however, the molecular mechanism underlying UCA1 involvement in GC warrants further investigation.

A recent study has reported that lncRNA containing miRNA binding sites can act as ceRNAs to regulate target mRNA levels 20. UCA1 has been shown to harbor the recognition sequence of many miRNAs to be involved in some tumors. UCA1 enhances cell proliferation and 5‐fluorouracil resistance by inhibiting miR‐204‐5p in colorectal cancer 21. UCA1 functions as an endogenous sponge by directly binding to miR‐485‐5p and down‐regulates the expression of matrix metallopeptidase 14 (MMP14) 22. UCA1 enhances cell proliferation, invasion, and G0/G1 cell cycle arrest in melanomas by sponging to miR‐507 targeting FOXM1 23. In the current study, we identified miR‐590‐3p as a direct target of UCA1 by luciferase activity assay. Furthermore, we observed that lower miR‐590‐3p expression was negatively associated with higher UCA1 expression in GC tissues. Moreover, we demonstrated that knockdown of UCA1 can upregulate the levels of miR‐590‐3p expression; however, increased UCA1 had an opposite effect in GC cells. We found that UCA1 promoted GC cell proliferation, cell colony formation, and invasion by negatively regulating miR‐590‐3p. Thus, our results revealed a novel regulated molecular target for UCA1 in GC.

**Figure 7:**
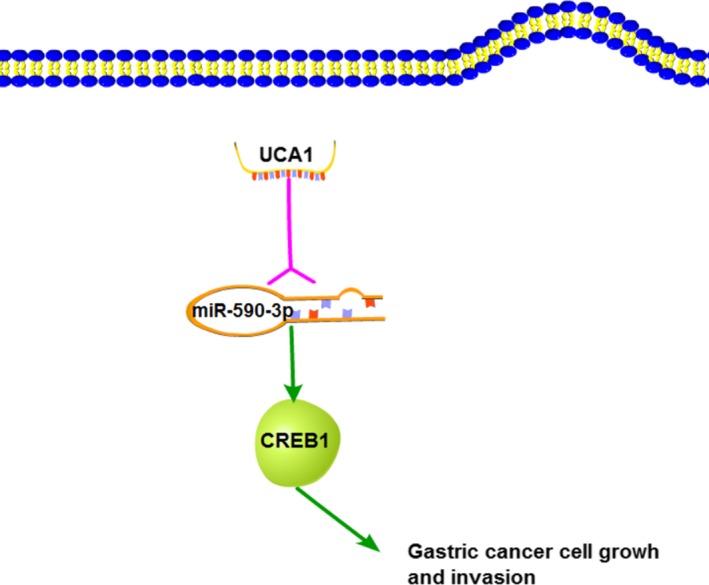
UCA1 promoted GC progression by sponging miR‐590‐3p targeting CREB1 in GC.GC, gastric cancer.

A previous report has indicated that CREB1 acts as an oncogene in GC; specifically, miRNA‐122 inhibits proliferation and invasion in gastric cancer by targeting CREB1 24. Knockdown of CREB1 inhibits tumor growth of human gastric cancer in vitro and in vivo 25. In the current study, using predictive tools and luciferase reporter gene assay analysis, we demonstrated that CREB1 is a target of miR‐590‐3p in GC cells. Furthermore, we showed that knockdown of UCA1 inhibits CREB1 expression, while co‐transfection of mir‐590‐3p inhibitor and si‐UCA1 reversed the decreased CREB1 expression induced by si‐UCA1. Thus, these results showed that CREB1 is a direct downstream target of UCA1.These results showed that UCA1 regulated CREB1 expression by sponging to miR‐590‐3p expression in GC cells (Fig. 7). Our findings revealed a UCA1/miR‐590‐3p/CREB1 axis involving GC cell proliferation and invasion.

In conclusion, we demonstrated that UCA1 is increased in GC and promotes cell proliferation, colony formation, and cell invasion by negatively regulating miR‐590‐3p expression. Moreover, we revealed that UCA1 activates CREB1 expression by sponging to miR‐590‐3p in GC cells. Thus, these results showed that inhibition of UCA1/miR‐590‐3p/CREB1 may serve as a target for GC treatment.

## Conflict of Interest

None declared.
